# The Pronunciation of “U”

**Published:** 1880-12

**Authors:** 


### THE PRONUNCIATION OF "U."

Ninety-nine out of every hundred Northerners will say institoot instead of institute, dooty for duty—a perfect rhyme to the word beauty. They will call new and news noo and noos—and so on through the dozens and hundreds of similar words. Not a dictionary in the English language authorizes this. In student and stupid, the "u" has the same sound as in cupid, and should not be pronounced stoodent and stoopid, as so many teachers are in the habit of sounding them.

It is a vulgarism to call a door a doah—as we all admit—isn't it as much of a vulgarism to call a newspaper a noospaper? One vulgarism is Northern, and the other Southern, that's the only difference. When the *London Punch* wishes to burlesque the pronunciation of servants, it makes them call the duke the dook, the tutor the tooter, and a tube a toob. You never find the best Northern speakers, such as Wendell Philips, George William Curtis, Emerson, Holmes, and men of that class, saying noo for new, Toosday for Tuesday, avenoo for avenue, or calling a dupe a doop. It is a fault that a Southerner never falls into. He has slips enough of another kind, but he doesn't slip on the long "u." As many of our teachers have never had their attention called to this, I hope they will excuse this notice.—*Southern Letter.*

---

Look at This and Then at That.—Miss Blanche Murray is a very proper young lady. Last week she caught her little brother smoking.

"You terrible thing," she hissed, "I am going to tell father."

"This is only corn silk," murmured the boy, penitently.

"I don't care what it is. I am going to tell on you, and see that you don't get into that beastly, horrid, degrading habit."

II.

It is evening. Miss Murray is sitting on the front stoop with Algernon. It is moonlight, and the redolent spirits of the honeysuckles and syringa are wafting bliss to their intoxicated souls.

"Would little birdie object to my smoking a cigarette?"

"Not at all," replied Miss Murray. "I like cigarettes. They are so fragrant and romantic. I think they are just too delicate for anything."

"Then I'll light one."

He lights a cigarette, and they talk about the weather for two hours and a half.—*Petroleum World.*

He Deserved It.—A good story is told of one of the grand jurors from Northfield, Vt., in attendance upon the last term of court at Montpelier. He was afraid he would not wake in season to take the six o'clock train for the capital, which he had talked over with his wife on retiring. He had just got into a sound sleep when his faithful spouse woke him, suggesting that it must be time to get up. He did ; found it was only midnight, and retired again. Soon he was again aroused, and this time, upon consulting the clock, it proved to be only 2 A. M. Somewhat disgusted and angered at being so often broken of his slumbers, he again sought his couch, admonishing his wife thus : "Look here, you keep your elbow out of my back, and your mouth out of my ear till morning." Feeling herself relieved of any further responsibility she went to sleep for good and left her lord and master to wake when he chose, which he did at four o'clock. Thinking it would not pay to try and get any more sleep, he built a fire, put on his overcoat and hat, took his valise in his hand and sat down before the fire for a few minutes. Meanwhile his wife slumbered on until seven o'clock, when she woke to find him gone, whereat she felt quite badly, saying she intended to have got him a warm breakfast. Leisurely proceeding to dress herself, she sought the kitchen, where, to her astonishment, she beheld her husband, sitting bolt upright in his chair before the stove, fast asleep, with the train gone over an hour.

---

The London *Church Times* tells the story of some Sunday-school children being taken for a picnic to the seaside. One of the teachers asked one of her scholars how she liked the sea. "Very much, miss," replied the child. "But where are the tinnamies?" "The tinnamies, my child? What do you mean?" "Why, you know," the child replied again, "the tinnamies that go down with the sea. You know the commandment says: 'The sea and all the tinnamies.'" The teacher was quite mortified to find that this was the way the child had been repeating : "The seas and all that in them is." We have known American children that wondered much about the contents of the mysterious miz, "the sea and all in the miz." But these mistakes are nothing to the true story of an inspector of religious knowledge in the diocese of Manchester, who discovered a child who, in reciting the Apostles' Creed, transformed "Suffered under Pontius Pilate" into "Suffered under bunch of spiders."

### THE IDEAL.

I think the song that's sweetest
Is the one that's never sung;
That lies at the heart of the singer
Too grand for mortal tongue.
And sometimes in the silence
Between the day and night,
He fancies that its measures
Bid farewell to the light.

A picture that is fairer
Than all that have a part
Among the masterpieces
In the marble halls of art,
Is the one that haunts the painter
In all his golden dreams,
And to the painter only
A real picture seems.

The noblest, grandest poem
Lies not in blue and gold
Among the treasured volumes
That rosewood bookshelves hold;
But in bright, glowing visions,
It comes to the poet's brain,
And when he tries to grasp it
He finds his effort vain.

A fairy hand from dreamland
Beckons up here and there,
And when we strive to clasp it
It vanishes into air.
And thus our fair ideal
Floats always just before,
And we with longing spirits
Reach for it evermore.

